# Anticoagulant properties of enoxaparin 400 IU/mL–40 % ethanol catheter lock solution

**DOI:** 10.1186/s40064-015-1533-2

**Published:** 2015-12-01

**Authors:** Laure Calvet, Michèle Piot, Claire Lartigue, Bertrand Souweine, Brigitte Tardy-Poncet

**Affiliations:** Service de Réanimation Médicale Polyvalente CHU de Clermont-Ferrand, Clermont-Ferrand, France; Université de Lyon, 42023 Saint-Etienne, France; Groupe de Recherche sur la Thrombose, EA 3065, 42023 Saint-Etienne, France; Laboratoire Chimie Analytique et Spectrométrie de masse UMR 990 Inserm-UdA, Clermont-Ferrand, France; Laboratoire Microorganismes: Génome Environnement (LMGE), Clermont Université, UMR CNRS 6023, Université d’Auvergne, Clermont-Ferrand, France; Laboratoire d’Hématologie, CHU Saint-Etienne, Hôpital Nord, 42055 Saint-Etienne, France; Hôpital Gabriel Montpied, Service de Réanimation Médicale Polyvalente, CHU de Clermont-Ferrand, 63003 Clermont-Ferrand Cedex 01, France

**Keywords:** Thrombin generation, Enoxaparin, Ethanol, Catheter lock, Lock solution, Antithrombotic effect

## Abstract

Unfractionated heparin (UFH) is the most widely used interdialytic lock solution but has no anti-infectious properties. Ethanol at a content ≥40 %v/v eradicates experimental biofilm but has no anticoagulant properties. In contrast to UFH, enoxaparin (Enox) can be combined with 40 % ethanol without precipitation. Enoxaparin 400 UI/mL–40 % ethanol (Enox/Eth) has antibiofilm properties and therefore has promise as an alternative lock solution. This study assessed the anticoagulant properties of Enox/Eth. Enox and Enox/Eth were diluted in whole blood at a final Enox concentration of 0.5, 1 (N = 6 samples), 1.5 (N = 4) and 2 (N = 6) IU/mL. Anti-Xa activity was determined by chromogenic assay and the inhibition of endogenous thrombin potential (ETP) by thrombinography. Quantitative data were compared by the Mann-Withney U test. For Enox concentrations of 0.5, 1, 1.5 and 2 UI/mL in whole blood samples, the mean ± SD values of the anti-Xa activity were 0.68 ± 0.09, 1.26 ± 0.14, 1.73 ± 0.30, 2.35 ± 0.32 UI/mL for Enox/Eth and 0.94 ± 0.15, 1.80 ± 0.22, 2.74 ± 0.23, 3.54 ± 0.44 UI/mL for Enox (P = 0.03, P = 0.03, P = 0.13, P = 0.03); and of the percentage of ETP inhibition was 17.36 ± 9.65, 30.27 ± 17.06, 36.5 ± 17.06, 57.82 ± 15.42 for Enox/Eth, and 42.96 ± 15.68, 68.93 ± 10.01, 83.5 ± 8.81, 91.19 ± 4.67 for Enox (P = 0.03, P = 0.03, P = 0.13, P = 0.03), respectively. The median and IQR values of Enox concentration inhibiting 50 % of ETP (IC50 ETP) were 1.8 [1.1–2.4] IU/mL for Enox/Eth and 0.7 [0.3–0.9] IU/mL for Enox, P = 0.03. Enox/Eth has strong anticoagulant activity, albeit lower than that of Enox, but with an extremely low IC50 ETP compared to the Enox concentration of non-diluted Enox/Eth.

## Background

Central venous catheters are increasingly used for long-term vascular access in end stage renal disease patients with limited access for hemodialysis. The main complications related to hemodialysis catheter use are infection and thrombosis. Interdialytic catheter locking with unfractionated heparin (UFH) is routinely performed to maintain catheter patency (Jain et al. 2009; Moran and Ash 2008). UFH has no anti-infectious properties and despite its widespread use as interdialysis lock solution, catheter infection remains a major complication entailing substantial morbidity, mortality, and additional costs. Antibiotic locking reduces the rate of catheter infection (Zhao et al. 2014) but is not recommended because it promotes bacterial resistance (Landry et al. 2010; Dixon et al. 2012).

Ethanol is a wide spectrum antimicrobial agent that acts by protein denaturation with little risk of the emergence of resistant organisms. A preliminary randomized controlled trial involving chronic dialysis patients suggested that 70 %v/v ethanol locking administered once a week could be effective in reducing catheter infection (Broom et al. 2012). Exposure of intravascular devices to high concentrated ethanol poses questions about its effect on the structural stability and the mechanical properties of catheters (Guenu et al. 2007; Msakni et al. 2013; Mermel and Alang 2014). In contrast, immersing catheters in 40 %v/v ethanol has only a marginal impact on catheter integrity (Msakni et al. 2013). No clinical study has evaluated the efficacy of 40 % ethanol lock in preventing catheter infection. However, ethanol at content ≥40 % (v/v) exerts antibiofilm effects against most microorganisms commonly involved in catheter infections (Balestrino et al. 2009; Lesens et al. 2013; Öncü 2014) and, therefore, 40 % ethanol could be an attractive antimicrobial agent for lock solution.

However, ethanol has no anticoagulant properties and so the optimal lock solution could be a mixture of heparin and ethanol. Unfortunately, UFH cannot be combined with 40 % ethanol because of precipitation (Lartigue et al. 2015). By contrast, low molecular weight heparins can be combined with 40 % ethanol depending on their concentrations, and of these, enoxaparin (Enox) exhibits the highest solubility in 40 % ethanol (Lartigue et al. 2015). A mixture of Enox 400 UI/mL–40 % ethanol (Enox/Eth) has an antibiofim activity similar to that observed with 40 % ethanol alone and has only a marginal impact on silicone and polyurethane catheter integrity (Balestrino et al. 2015). Thus, Enox/Eth could be an attractive alternative interdialytic lock solution. Whether Enox/eth has persistent anticoagulant properties, however, remains unknown. The purpose of this study was to assess the anticoagulant activity of Enox/eth.

## Methods

### Mixtures

The solutions were prepared on the day before the test. Enox 400 IU/mL was prepared with Enox sodium (Lovenox^®^ 4000 IU/0.4 ml, Sanofi-Aventis, France) diluted in 0.9 % sodium chloride (Aguettant, Lyon, France), 40 %v/v ethanol with 99 % ethanol (Carlo Erba, Peypin, France) diluted in 0.9 % sodium chloride, and Enox/Eth with Enox 400 IU/mL diluted in ethanol and 0.9 % sodium chloride. The density of Enox/Eth determined at room temperature (23 °C) by specific gravity bottle (pycnometer) according to the European Pharmacopeia recommendations (6.0 (01/2008:20205) was 0.9498.

On the day of the test, venous blood from 6 blood donors, 4 women and 2 men, was collected into Vacutainer tubes (Becton–Dickinson, Meylan Cedex, France) containing 0.129 M sodium citrate (1 vol anticoagulant and 9 vol whole blood). The first 2–3 ml of whole blood, which could potentially be contaminated by tissue factor present in the skin or vascular cells, were discarded. The samples were analyzed within 2 h of collection as recommended.

Enox 400 IU/mL was added to whole blood to obtain blood samples with enoxaparin concentrations of 0.5, 1, 1.5 and 2 IU/mL; 40 % ethanol was added to whole blood to obtain blood samples with ethanol contents of 0.05, 0.1, 0.15 and 0.2 %v/v; and Enox/Eth was added to whole blood to obtain blood samples with enoxaparin concentrations/ethanol contents of 0.5/0.05, 1/0.1, 1.5/0.15 and 2/0.2 IU/mL/ %v/v. The control was prepared by mixing 0.9 % sodium chloride with whole blood.

All mixtures were prepared in polypropylene tubes and then incubated 1 h at 37 °C with gentle shaking every 10 min to mimic in vivo conditions of catheter lumen. They were subsequently centrifuged at 200xg, for 10 min at 20 °C to obtain platelets rich plasma (PRP), which were immediately used for thrombinography assays. Platelet poor plasma (PPP) were obtained by centrifugation (twice at 2500*g* for 15 min) and stored at −80 °C until anti-Xa assays.

### Anticoaguant activity

The anticoagulant properties were assessed by measurement of both antithrombotic activity and anti Xa activity.

### Antithrombotic activity

All assays were performed in triplicate. Antithrombotic activity was determined by measurement of thrombin generation (TG) by thrombinography as described elsewhere (Tardy-Poncet et al. 2009). Recombinant human tissue factor (Innovin) was purchased from Dade Behring (Marburg, Germany), fluorogenic substrate, Z-Gly-Gly-Argaminomethylcoumarin (Z-GGR- AMC) from Bachem (Weil am Rhein, Germany), thrombin calibrator from Diagnostica Stago (Asnières, France), 96-round bottom well microplates from Dutscher Greiner (Brumath, France) and Bovine serum albumin (BSA) from Sigma (St Quentin Fallavier, France). Thrombogram software was supplied by Synapse (Maastricht, The Netherlands).

Twenty microlitre of diluted tissue factor (final dilution 1/1200) in HBS buffer (Hepes 20 mM, NaCl 140 mM, BSA 5 g/L, pH 7.35) were dispensed into the wells. Twenty µL of thrombin calibrator were added to the calibrant wells. Eighty µL of each fresh PRP mixture were then placed in the wells. The plate was inserted into a Fluoroskan Ascent plate reader (Thermolab Systems, Helsinki, Finland) and preheated to 37 °C for 10 min. Coagulation was triggered by the automated addition of 20 µL of Z-GGR-AMC (2.5 mM) dissolved in 20 mM Hepes buffer (pH 7.35) containing 0.1 M CaCl2 and 60 g. L-1 BSA. The plate was then shaken for 12 s. Fluorescence intensity was determined at wavelengths of 390 nm (excitation filter) and 460 nm (emission filter) every 15 s during 60 min. TG was monitored continuously using the calibrated thrombogram method described by Hemker et al. (2003), in which the initial derivative curves of fluorescence accumulation are converted into TG curves using a human thrombin calibrator and Thrombinoscope software (version 3.0.25; Biodis, Signes, France).

Thrombinography was performed on PRPs containing 40 % ethanol, Enoxaparin, and Enox/Eth. The following thrombogram parameters were measured: lag-time (LT, min), thrombin peak (TP, nmol thrombin), endogenous thrombin potential (ETP, nmol*min) which corresponds to the area under the curve, time to peak (ttP, min) and velocity index, which corresponds to the propagation phase of thrombin generation calculated by the formula TP/(ttP-LT). The anti-thrombotic effect of the different mixtures was expressed in percentage of ETP inhibition (ETP(basal) − ETP (solution tested)/ETP(basal) × 100). IC50 ETP was defined as the concentration of a solution inhibiting 50 % of ETP and represents classically the concentration required to achieve adequate anticoagulation (al Dieri et al. 2004).

### Anti-Xa activity

Enox anti-Xa activity was measured on PPP. The assays were performed on an automated coagulometer BCS (Siemens, France), using an anti-Xa chromogenic assay (Biophen Heparin, Hyphen BioMed, Neuville sur Oise, France) according to the specific recommendations of Hyphen Biomed. The persistency of the anti-Xa activity of Enox 400 UI/ml and Enox/Eth at the final Enox concentration of 1 IU/mL was assessed in one donor PPP at 30 min, 23 h, 46 h, 119 h and 216 h.

### Statistical analysis

Results were expressed as mean ± standard deviation (SD) or median and interquartile ranges (IQR). Quantitative data of the different solutions were compared by the Man-Withney U test, and anti Xa activity and ETP inhibition by Spearman correlation. Statistical analyses were carried out with Statview 5.0 software (SAS Institute, Cary, NC, USA). A p value ≤0.05 was considered statistically significant.

### Ethics statement

Human blood samples used in this study came from 6 healthy donors at the local French Blood Service [Etablissement Français du Sang, Saint-Etienne]. After being fully informed as required by the Public Health Code (article R.1221-5, decrees of 01/12/2009 and 06/11/2006), all donors involved in our study gave written consent.

## Results

The mean basal value of ETP in the controls was 1730 ± 325 nmol*min. TG measured in PRP from whole blood samples at ethanol contents of 0.05, 0.1, 0.15 and 0.2 %v/v was similar to TG in controls, demonstrating that ethanol alone at these low concentrations has no impact on the antithrombotic activity (data not shown).

**Fig. 1:**
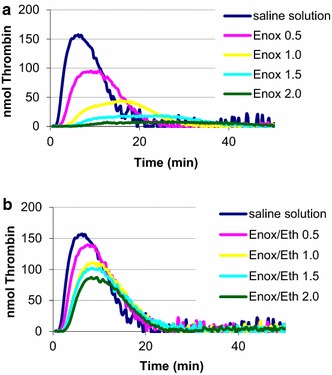
Representative thrombin generation curves from an individual blood donor measured in PRP, after whole blood incubation for 1 h à at 37 °C with: **a** Enox (blood concentration 0.5–2.0 IU/m/L), and **b** Enox/Eth (blood concentration 0.5–2.0 IU/m/L) Thrombogram parameters—Lag-time (min): initiation phase of thrombin generation; Peak: maximal concentration (Cmax) of thrombin generated, expressed in nM; Time to peak (TtPeak time in min) necessary to achieve maximal thrombin concentration (Tmax); Endogenous thrombin potential (ETP): (nMxmin) area under the curve (represents thrombin molecule activity in plasma); Start-Tail: time (min) to at the term of which the curve of the thrombogram returns to zero

**Fig. 2:**
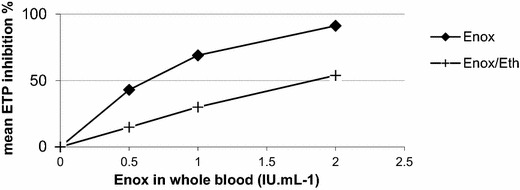
Mean IC 50 ETP of Enox (*diamond symbols*) and Enox/Eth (*cross symbols*) from 6 blood donors

**Fig. 3:**
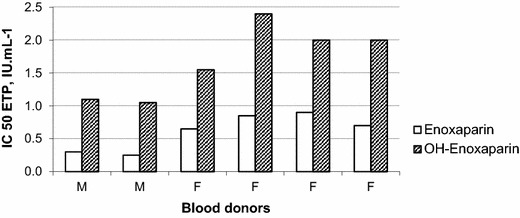
IC50 ETP of Enox/Eth and Enox: inter-individual and sex related variability. *M* men donors, *F* women donors

Enox/Eth at Enox concentrations between 0.5 IU/mL and 2.0 IU/mL was associated with a 15–54 % ETP inhibition (Table 1, Fig. 1). For each Enox concentration, ETP inhibition of Enox/Eth was lower than that observed with Enox (Fig. 2). IC50 ETP median and IQR values were 1.8 [1.1–2.4] IU/mL for Enox/Eth and 0.7 [0.3–0.9] IU/mL for Enox, P = 0.03. Lower ETP inhibition values and therefore higher IC 50 ETP values were observed in females than in males for both Enox/Eth and Enox (Fig. 3).

**Table 1:** Results of the inhibition of endogenous thrombin potential (ETP) induced by enoxaparin (Enox) and the mixture of enoxaparin 400 IU/mL–40 % ethanol (Enox/Eth) measured on platelet rich plasma by thrombinography according to Enox concentration in whole blood from donors

Enox (IU/mL)^a^	ETP inhibition of Enox^b^	ETP inhibition of Enox/eth^b^	P value
0.5 (N = 6)	42.96 ± 15.66	17.36 ± 9.65	0.03
1 (N = 6)	68.93 ± 10.01	30.27 ± 17.06	0.03
1.5 (N = 4)	83.5 ± 8.81	36.5 ± 17.06	0.13
2 (N = 6)	91.19 ± 4.67	57.82 ± 15.42	0.03

*Enox* enoxaparin, *Enox/Eth* enoxaparin 400 IU/mL–40 % ethanol

^a^Concentration in whole blood

^b^In percentage and expressed as mean ± standard deviation

The anticoagulant effect of Enox in Enox/Eth was demonstrated with the Anti-Xa tests in all PPP samples with a correlation between whole blood Enox concentration and anti-Xa activity (Table 2). For each whole blood Enox concentration, there was a mean decrease of 26.7–36.7 % in the anti-Xa activity of PPP with Enox/Eth as compared to PPP with Enox alone. The anti-Xa activity ratio of PPP with Enox to PPP with Enox/Eth was stable over time: 1.02 at 30 min, 0.83 at H23, 0.82 at H 46, 0.85 at H 119, and 0.82 at H 216 (one sample tested).

**Table 2:** Results of the Anti Xa activity induced by enoxaparin (Enox) and the mixture of enoxaparin 400 IU/mL–40 % ethanol (Enox/Eth) measured on platelet rich plasma by thrombinography according to Enox concentration in whole blood from donors

Enox (IU/mL)^a^	Anti Xa activity of Enox^b^	Anti Xa activity of Enox/eth^b^	P value
0.5 (N = 6)	0.94 ± 0.15	0.68 ± 0.09	0.03
1 (N = 6)	1.80 ± 0.22	1.26 ± 0.14	0.03
1.5 (N = 4)	2.74 ± 0.23	1.73 ± 0.31	0.13
2 (N = 6)	3.54 ± 0.44	2.35 ± 0.32	0.03

*Enox* enoxaparin, *Enox/Eth* enoxaparin 400 IU/mL–40 % ethanol

^a^Concentration in whole blood

^b^In IU/mL and expressed as mean ± standard deviation

**Fig. 4:**
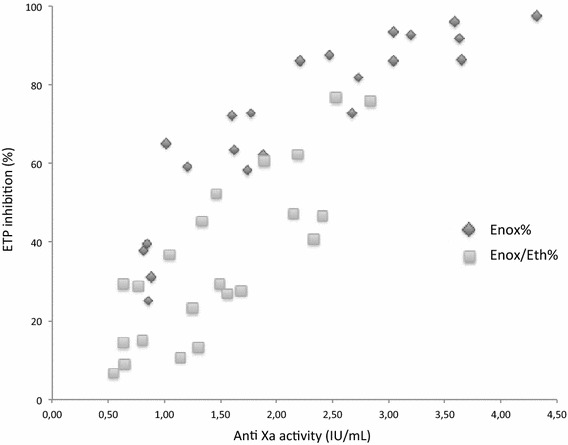
Inhibition of the endogenous thrombin potential (ETP) as a function of Anti Xa enoxaparin activity in poor platelet plasma

We further investigated the relation between anti-Xa activity and ETP. Plotting ETP inhibition against anti-Xa activity showed a strong relation between the increase in ETP inhibition and the increase in the anti-Xa activity for Enox/Eth (P = 0.04) and Enox alone (P < 0.0001), (Fig. 4). For Enox alone, there was a sharp increase in inhibition at low Enox activity that approached complete inhibition when the anti-Xa activity was >2.5 IU/mL. For Enox/Eth, Fig. 4 displays a similar pattern, suggesting a hyperbolic relationship between ETP and anti-Xa activity and complete inhibition, but at higher values than those observed for Enox alone.

## Discussion

Our study adds important information regarding the anticoagulant properties of Enox/Eth by demonstrating both its antithrombotic and anti-Xa activity.

Most studies on the in vitro properties of catheter lock solutions containing both injectable anticoagulants and antimicrobials have focused on mixtures of heparin with antibiotics, and are limited to their chemical stability and antimicrobial properties (Krishnasami et al. 2002; Droste et al. 2003; Anthony and Rubin 1999; Vercaigne et al. 2000, 2002; Capdevila et al. 2001). The stability of Enox/Eth may be questionable since mixing ethanol with heparin may result in heparin precipitation. The assessment of Enox/Eth solubility by visual observation showed that the mixture remained clear for up to 72 h of incubation at room temperature (23 °C) and at 37 °C. The stability of Enoxaparin 400 IU/mL and that of 40 % ethanol in Enox/Eth was confirmed by the HPLC-ELSD method and by a titrimetric dichromate assay, respectively (Lartigue et al. 2015). In vitro studies have shown that high concentrated ethanol exposure induces changes in the structural stability of polyurethane catheters and to a lesser extent that of silicone catheters (Guenu et al. 2007; Msakni et al. 2013) and may result in catheter occlusion and breaches (Mermel and Alang 2014). However, immersing catheters in 40 % ethanol or in Enox/Eth has only a marginal impact on polyurethane catheter integrity (Msakni et al. 2013; Balestrino et al. 2015), which suggests that Enox/Eth ethanol has only a minimal effect on the mechanical properties of catheters.

Ethanol solutions at a concentration above 28 % can be associated with plasma protein precipitation (Schilcher et al. 2013). In our in vitro study, we did not assess the impact of Enox/Eth on plasma protein in a clinical setting and we did not analyze the spillage of Enox/Eth or the leakage of potentially precipitated protein particles into the systemic circulation. However, a previous experimental study showed that heparins inhibit the precipitation of plasma proteins (Pemberton et al. 2010). In addition, Enox/Eth has a lower density than blood and plasma (Trudnowski and Rico 1974), which suggests that Enox/Eth leakage into the systemic circulation followed by the entry of blood into the catheter would be negligible, particularly for catheters inserted at the jugular sites. Taken together, these data suggest that marked plasma protein precipitation due to spillage of Enox/Eth is unlikely.

The anticoagulant activity of these catheter lock solutions has been scarcely reported and mainly assessed by clotting tests (Robinson et al. 2005; Cullis et al. 2015). However, clotting time assays only probe the initiation phase of coagulation by measuring the time before the burst of thrombin starts and generates clot. The determination of the amount of thrombin activity that develops could be a better marker of the function of the clotting system. Thrombinography is a global test that measures the ability of a plasma sample to generate thrombin after in vitro initiation of coagulation. It reflects the initiation, propagation and termination phases of the coagulation, and therefore takes into account the pro and anticoagulant reactions governing thrombin formation. It is a suitable assay to measure the anticoagulant activity of anticoagulant drugs and particularly that of enoxaparin (al Dieri et al. 2004; Hemker et al. 2002; Gerotziafas et al. 2004; Robert et al. 2009). ETP is the area under the thrombin generation curve and is considered the most robust parameter of the thrombogram (TG curve) for estimating the total amount of thrombin formed over time (Al Dieri et al. 2012). To our knowledge, our study is the first to use thrombinography to assess the antithrombotic effect of a lock solution.

Numerous studies have assessed the hemostatic properties of ethanol owing to the association between light to moderate alcohol drinking and a lower incidence of ischemic heart disease resulting from suppression of thrombus formation and atherosclerotic progression, and a higher bleeding tendency (Mukamal et al. 2003). Ethanol inhibits platelet aggregation and impairs fibrinolysis (Marumo and Wakabayashi 2010; Engström et al. 2006; Ehrlich and Humpel 2014). The results of studies reporting the impact of ethanol on the coagulation vary according to the experimental conditions and the tests used (Engström et al. 2006; Bloemen et al. 2012). In preliminary tests, we found that ethanol at contents ranging between 0.05 and 0.2 %v/v had no effect on TG parameters either at room temperature or at 37 °C. Similar results have already been published (Bloemen et al. 2012). In contrast, we found that when ethanol is mixed with Enox at Enox concentrations/ethanol contents of 0.5/0.05, 1/0.1, 1.5/0.15 and 2/0.2 IU/mL/%v/v, ethanol diminishes the antithrombotic effect of enoxaparin by two-thirds by decreasing IC50 ETP from 1.8 IU.mL-1 for Enox/Eth to 0.7 IU.mL-1 for Enox alone. The cause of the diminished antithrombotic effect of Enox when Enox is mixed with ethanol is speculative, but we cannot exclude that platelet exposed to ethanol shed PF4 results in a decrease in enoxaparin activity.

In our study we observed an inter-individual variability in the antithrombotic effect of Enox and Enox/Eth. Large variation in TG response to heparin between individuals and low molecular weight heparin for the same anti-Xa level is classically reported and probably reflects inter-individual variability in baseline coagulation potential (Cullis et al. 2015; Hemker et al. 2002; Chowdary et al. 2015). We also observed lower ETP inhibition in females than in males in response to both Enox and Enox/Eth. Differences in TG values according to gender, with higher ETP in women, have already been reported (Marchi et al. 2015). Lower susceptibility of women to heparin has been suggested by in vitro experiments using thromboelastography (Monte and Lyons 2004).

The decreased antithrombotic effect of Enox/Eth compared to that of Enox alone and the gender variability that we observed have probably only a marginal impact on the anticoagulant properties of Enox/Eth, since Enox concentration in Enox/Eth is more than 200-fold higher than the concentration used in our experiments.

The dilutions of Enox used in this study are representative of clinical practice. When measuring Enox anti-Xa activity in PPP, we found higher values than in whole blood. This makes sense because the volume of blood is larger than the volume of PPP, and the total amount of Enox in both whole blood and PPP was equivalent (very little Enox being sequestrated in red blood cells). The results of the anti Xa tests shows that for each Enox concentration, the anti-Xa activity of Enox/Eth was decreased by approximately one-third as compared to Enox alone. As mentioned above in the TG results, the discrepancy in the anti-Xa activity between Enox alone and Enox/Eth is probably only marginal because of the extreme dilution of Enox/Eth in the samples. Furthermore, the decrease in the anti-Xa activity of Enox/Eth was observed in the first 24 h, with no further decrease up to 216 h afterwards, suggesting that Enox/Eth instilled in a catheter for a dwell time of 48 to 72 h could exert its anti-Xa activity during the entire interdialytic period.

Our study presents some limitations. First, the antithrombotic properties of Enox/Eth were investigated in a small sample of 6 Caucasians (4 women and 2 men) and revealed inter-individual variability in the antithrombotic effect but all individual results follow the same trend and show an anticoagulant effect of the mixture. However, whether the results can be applied to a non-Caucasian population is speculative and would require further large-scale studies. Second, blood used in the study was obtained from healthy adult donors, and not from chronic dialysis patients. Possible alterations in the anticoagulant status of end stage renal disease patients might affect hemostasis tests and yield different results. However, previous studies using thrombinography reported the presence of hypocoagulability in chronic dialysis patients (Jeong et al. 2013; Brophy et al. 2006), which suggests that the strong anticoagulant activity of Enox/Eth observed with blood from healthy adult donors would not have been diminished if we had used blood from chronic dialysis patients. Third, as is standard in this kind of test, the experiments were performed using polypropylene tubes, whereas in a clinical setting the lock solutions are instilled into polyurethane or silicone catheters. We cannot exclude that the anticoagulation properties of lock solutions observed in the polypropylene tubes may be modified when the solution is exposed to other materials. Fourth, the conditions of this in vitro-study differ widely from those of the clinical setting. In particular, they do not take into account the seepage of the lock solution into the systemic circulation, which is accompanied by a concomitant blood inflow into the catheter. The leakage of catheter lock solutions may dilute the lock solution and favor plasma protein precipitation by the high ethanol content contained in Enox/Eth (Schilcher et al. 2013).

## Conclusion

In conclusion, Enox/Eth exerts strong anticoagulant activity. We previously demonstrated the stability of Enox/Eth (Lartigue et al. 2015), its low impact on polyurethane and its antibiofilm activity against the microorganisms commonly involved in catheter infections (Balestrino et al. 2015). Taken together these findings suggest that Enox/Eth may be a suitable catheter lock solution for preventing catheter infection and maintaining catheter patency. A large randomized control trial is now warranted to assess the efficacy of Enox/Eth in preventing dialysis catheter infection in chronic hemodialysis patients.

## Abbreviations

ETP: endogenous thrombin potential
Enox: enoxaparin
Enox/Eth: enoxaparin 400 UI/mL–40 % ethanol
IC50 ETP: concentration of a solution inhibiting 50 % of ETP
IQR: interquartile ranges
LT: lag-time
PPP: platelets poor plasma
PRP: platelets rich plasma
SD: standard deviation
TG: thrombin generation
TP: thrombin peak
UFH: unfractionated heparin
